# Deciphering the Role of Pyrvinium Pamoate in the Generation of Integrated Stress Response and Modulation of Mitochondrial Function in Myeloid Leukemia Cells through Transcriptome Analysis

**DOI:** 10.3390/biomedicines9121869

**Published:** 2021-12-09

**Authors:** Yu-Hsuan Fu, Chi-Yang Tseng, Jeng-Wei Lu, Wen-Hui Lu, Pei-Qi Lan, Chien-Yuan Chen, Da-Liang Ou, Liang-In Lin

**Affiliations:** 1 Department of Clinical Laboratory Sciences and Medical Biotechnology, National Taiwan University, Taipei 100229, Taiwan; b02404009@ntu.edu.tw (Y.-H.F.); d08424002@ntu.edu.tw (C.-Y.T.); ff235841@gmail.com (W.-H.L.); peggylan0421@gmail.com (P.-Q.L.); 2 Antimicrobial Resistance Interdisciplinary Research Group, Singapore-MIT-Alliance for Research and Technology, Singapore 138602, Singapore; jengweilu@gmail.com; 3 Division of Hematology, Department of Internal Medicine, National Taiwan University Hospital, Taipei 100225, Taiwan; chienyuanchen@ntu.edu.tw; 4 Department of Oncology, National Taiwan University, Taipei 100025, Taiwan; 5 Department of Laboratory Medicine, National Taiwan University Hospital, Taipei 100225, Taiwan

**Keywords:** acute myeloid leukemia, *FLT3*-ITD, pyrvinium pamoate, integrated stress response

## Abstract

Pyrvinium pamoate, a widely-used anthelmintic agent, reportedly exhibits significant anti-tumor effects in several cancers. However, the efficacy and mechanisms of pyrvinium against myeloid leukemia remain unclear. The growth inhibitory effects of pyrvinium were tested in human AML cell lines. Transcriptome analysis of Molm13 myeloid leukemia cells suggested that pyrvinium pamoate could trigger an unfolded protein response (UPR)-like pathway, including responses to extracellular stimulus [*p*-value = 2.78 × 10^−6^] and to endoplasmic reticulum stress [*p*-value = 8.67 × 10^−7^], as well as elicit metabolic reprogramming, including sulfur compound catabolic processes [*p*-value = 2.58 × 10^−8^], and responses to a redox state [*p*-value = 5.80 × 10^−5^]; on the other hand, it could elicit a pyrvinium blunted protein folding function, including protein folding [*p*-value = 2.10 × 10^−8^] and an ATP metabolic process [*p*-value = 3.95 × 10^−4^]. Subsequently, pyrvinium was verified to induce an integrated stress response (ISR), demonstrated by activation of the eIF2α-ATF4 pathway and inhibition of mTORC1 signaling, in a dose- and time-dependent manner. Additionally, pyrvinium could co-localize with mitochondria and then decrease the mitochondrial basal oxidative consumption rate, ultimately dysregulating the mitochondrial function. Similar effects were observed in cabozantinib-resistant Molm13-XR cell lines. Furthermore, pyrvinium treatment retarded Molm13 and Molm13-XR xenograft tumor growth. Thus, we concluded that pyrvinium exerts anti-tumor activity, at least, via the modulation of the mitochondrial function and by triggering ISR.

## 1. Introduction

Acute myeloid leukemia (AML) is a heterogeneous hematological malignancy, characterized by the abnormal proliferation and differentiation blockage of progenitor stem cells, leading to leukemogenesis [1]. The past decades have witnessed substantial progress in the biological classification of AML [2]. Drug therapy for AML underwent significant advances in 2017 and 2018, with the US Food and Drug Administration (FDA) approving several new targeted agents [3,4]. Among these, midostaurin [5] and gilteritinib [6] were reported to be suitable for AML patients with a fms-like tyrosine kinase 3 (*FLT3*) mutation. The use of these specific FLT3 inhibitors alone exhibited relatively high response rates (50–66%); however, the duration of responses and survival were found to be brief, with patients living just 20–33 weeks [7]. Therefore, the development of more effective and long-lasting treatment strategies is warranted, especially for AML patients with the *FLT3* mutation, which is among the most common mutations in AML [8]. In recent years, researchers have attempted to use established, FDA-approved drugs, to help devise appropriate treatment options.

Pyrvinium pamoate is an FDA-approved anthelminthic drug used for the treatment of pinworms in humans [9]. The treatment of pinworm infections in humans (enterobiasis) with pyrvinium pamoate was reported as early as 1959 [10]. Recent studies have shown that pyrvinium selectively inhibits the growth of tumor cells of diverse tissue origins, including pancreas, colon, breast, brain, myeloma, and other hematological malignancies, without affecting the normal, healthy cells of the body [11,12,13]. The mechanism of the action of pyrvinium appears to vary in different tumor types. The following mechanisms have been elucidated: (1) through the allosteric activation of casein kinase 1α and the destabilization of β-catenin in wingless-related integration site (Wnt) pathways [14,15], as well as related Hedgehog signaling pathways [16]; (2) through the inhibition of the phosphoinositide 3-kinase (PI3K)/protein kinase B (AKT) signaling pathway [17]; (3) through the suppression of mitochondrial respiratory complex I or II [12,18,19], and the regulation of signal transducer and activator of transcription 3 (STAT3) activation [12]; (4) by targeting the unfolded protein response (UPR) during hypoglycemia [20]; (5) by targeting autophagy addiction [21]; and (6) by acting as a non-competitive androgen receptor inhibitor [22,23]. Of note, several studies exploring drug-repurposing have also reported pyrvinium as a potential anti-cancer compound [17,24,25,26,27,28]. However, the anti-cancer effect of pyrvinium on AML, and the underlying mechanisms, remain unclear.

Therefore, this study aimed to evaluate the in vitro and in vivo effects of pyrvinium in Molm13 leukemia cells, and conduct transcriptomic analysis to elucidate the mechanism through which it acts. Additionally, we also evaluated the effects of pyrvinium against a cabozantinib-resistant Molm13 cell line (Molm-13-XR).

## 2. Materials and Methods

### 2.1. Cell Culture and Chemicals

The human AML cell lines Molm13 (DSMZ, Brauschweig, Germany) and MV4-11 (DSMZ) were maintained in Roswell Park Memorial Institute (RPMI)-1640 medium (ThermoFisher Scientific/Gibco, Waltham, MA, USA), containing 10% fetal bovine serum (FBS); Kasumi-1 cells (ATCC, Manassas, VA, USA) were cultured in RPMI-1640 medium containing 20% FBS. The cabozantinib-resistant cell line, Molm13-XR, was established by culturing Molm13 cells in the presence of increasing concentrations of cabozantinib. The stable resistant cell line was then maintained in a complete RPMI-1640 medium with 10% FBS, in the absence of cabozantinib. Cell lines were cultured at 37 °C in a humidified atmosphere containing 5% CO_2_. The genetic profiles of these cell lines were authenticated (16-markers short tandem repeat) by the Food Industry Research and Development Institute (Hsinchu, Taiwan), and were found to be identical to their reported generic profiles.

Cabozantinib-malate, pyrvinium pamoate, and rapamycin were purchased from Selleck Chemicals (Houston, TX, USA), Sigma-Aldrich/Merck (Darmstadt, Germany), and TargetMol (Boston, MA, USA), respectively.

### 2.2. Cell Viability

The cells were exposed to the indicated doses of various compounds for 72 h, and the cell viability was measured using the Cell Titer 96 AQueous One Solution Cell Proliferation Assay (MTS assay; Promega, Madison, WI, USA), as per the manufacturer’s instructions. The half maximal inhibitory concentration (IC_50_) was determined using the CalcuSyn software (ver. 2.11; Biosoft, Cambridge, UK).

### 2.3. Cell Cycle Analysis

After incubating for 24 or 72 h with the drugs, the cells were harvested and washed with ice-cold phosphate buffered saline (PBS). The washed cells were then added to ethanol (Sigma-Aldrich/Merck, Darmstadt, Germany) while slowly vortexing, and were frozen at −20 °C for at least 24 h. The fixed cells were washed twice with PBS, stained with propidium iodide (PI; BD Biosciences, Franklin Lakes, NJ, USA) for 15 min and analyzed using a CytoFLEX flow cytometer (Beckman Coulter, Brea, CA, USA).

### 2.4. Immunoblotting

As previously described [29], protein lysates were separated in 10% polyacrylamide gel, and transferred to a polyvinylidene fluoride membrane. After blocking the non-specific binding sites, membranes were incubated with primary antibodies (Supplementary Table S1) at 4 °C overnight. Subsequently, membranes were washed three times with Tris-buffered saline containing polysorbate 20 (TWEEN 20) and incubated with horseradish peroxide-conjugated secondary antibodies (Cell Signaling Technology, Beverly, MA, USA) at room temperature for 1 h. Chemiluminescence signals were then generated by Western Lightening Plus-ECL (Perkin Elmer, Waltham, MA, USA) and captured by LAS4000 (Fujifilm, Tokyo, Japan). Quantification of protein and phosphorylation levels was performed by ImageJ (1.51 K).

### 2.5. Transcriptomic Analysis of Pyrvinium-Treated Molm13 Cells

Total RNA was isolated from the cells using the NucleoSpin^®^ RNA kit (Macherey-Nagel, Düren, Germany), after treatment with dimethyl sulfoxide (DMSO) or 100 nM pyrvinium for 6 h. The viability of the Molm13 cells collected for RNA extraction was more than 90% after this treatment. The RNA (1 μg) was then used for library preparation. A strand-specific RNA library was built using TruSeq stranded mRNA library prep Kit (cat# RS-122-2101, Illumina, San Diego, CA, USA), as per the manufacturer’s protocol. The libraries were then sequenced using the Illumina NovaSeq 6000 platform and 75 bps paired end reads were generated (Genomics, Taipei, Taiwan).

Sequencing alignment with hg19 was then performed using Rsubread (Bioconductor) [30], and differentially expressed genes (DEGs) analysis in the DMSO and pyrvinium groups was performed using EBSeq (Bioconductor) [31]. A threshold of ≥2-fold (or ≤0.5-fold) and *p*-value of ≤0.05 were set and used to screen DEGs. The transcriptome files can be found in the Gene Expression Omnibus (GEO) database (ID: GSE153854). Pathway enrichment was then analyzed by gene set enrichment analysis (GSEA) [32] using the Molecular Signatures Database v7.0 (MSigDB, Broad Institute, Cambridge, MA, USA) and Metascape [33]. The expression of genes was validated by quantitative reverse transcription-PCR (q-RT-PCR), according to methods previously described [29]. The primers used for specific gene detection are listed in Supplementary Table S2.

### 2.6. Confocal Microscopy Observation

After drug exposure for 24 h, PBS-washed cells were re-suspended in serum-free RPMI 1640 medium containing 100 μM MitoTracker Green (ThermoFisher Scientific/Gibco) and incubated for 30 min in a culture incubator. Mitochondria-stained cells were centrifuged again and stained with 3 μM of 4,6-diamidino-2-phenylindole (DAPI) for 15 min. Finally, the cells were washed again with serum-free medium and re-suspended in PBS. Cell suspensions were dropped on a glass slide and observed by confocal microscopy. The images of Confocal Microscopy were acquired by Zeiss LSM 880 and analyzed by ZEN software (Carl ZEISS, Jena, Germany).

### 2.7. Cellular Bioenergetic Analysis

The oxygen consumption rate (OCR) and extracellular acidification rate (ECAR) were analyzed in real-time using the XFe24 extracellular flux analyzer (Agilent Technologies/Seahorse Bioscience, Billerica, MA, USA), as per the manufacturer’s protocol. After the indicated drug treatment for 24 h, cells were seeded in the wells (1 × 10^5^ cells/well) of a 24-well microplate, precoated with Cell-Tak Cell and Tissue Adhesive (Corning, Corning, NY, USA). The Cell Mito Stress Test kit (Agilent Technologies/Seahorse Bioscience) was used for the measurement of mitochondrial respiration. After determination of the baseline respiration, oligomycin A, carbonyl-cyanide p-trifluoromethoxyphenylhydrazone (FCCP), rotenone and antimycin A were sequentially applied on the microplate to determine adenosine triphosphate (ATP)-linked respiration, protein leak, maximal respiratory capacity, and reserve capacity. The Glycolysis Stress Test kit (Agilent Technologies/Seahorse Bioscience) was used to assess the glycolytic activity in the cells following a sequential addition of glucose, oligomycin, and 2-deoxglucose (2-DG) in order to examine glycolysis, glycolytic capacity, and glycolytic reserve. The results were analyzed by Seahorse XFe24 wave software 2.2 (Agilent Technologies/Seahorse Bioscience).

### 2.8. Mitochondrial Parameters (Mitochondrial Mass and Reactive Oxygen Species (ROS) Levels)

The mitochondrial mass and ROS levels in cells were measured by flow cytometer (CytoFLEX flow cytometer, Beckman Coulter) using MitoTracker Green FM (Invitrogen/ThermoFischer Scientific, Waltham, MA, USA) and dichloro-dihydro-fluorescein diacetate (DCFH-DA; Sigma-Aldrich, St. Louis, MI, USA), respectively. To evaluate the mitochondrial mass in the living cells, drug-treated cells (1 × 10^6^) were re-suspended in RPMI -1640 serum-free medium containing 100 nM MitoTracker Green and incubated for 30 min under growth conditions. To determine the ROS levels, cells were stained with 10 μM DCFH-DA for 30 min at 37 °C in the dark, washed, and re-suspended in PBS.

### 2.9. Mitochondrial Complex I Activity

The mitochondria complex I activity was determined using the complex I enzyme activity microplate assay kit (Abcam, Cambridge, UK), as per the manufacturer’s instruction. The cells were exposed to the drugs for 24 h before being harvested, washed, and re-suspended in PBS. The protein concentration of the pellets was determined using the Bradford protein assay kit (Bio-Rad Laboratories, Hercules, CA, USA). The pellets were diluted to 5.5 mg/mL with PBS. Cells were then lysed with 10× detergent provided in the kit and diluted with incubation solution. Finally, 200 μg protein lysate was added to the 96-well microplate and incubated for 3 h. It was then washed thrice with a buffer solution. After the assay solution was added, A_450_ absorbance signals were detected kinetically every 20 s for 30 min using a Spectra Max M5 microplate reader (Molecular Devices).

### 2.10. Subcutaneous Mouse Xenografts

To assess the efficacy of pyrvinium pamoate in vivo, a mouse subcutaneous xenograft model was established in Female CAnN.Cg-*Foxn1*^nu^/CrlNarl (nude) mice. Nude mice aged 6–8 weeks were purchased from the National Laboratory Animal Center, NARLabs, Taiwan. The mice were anesthetized by inhaling isoflurane and maintained with 2% isoflurane, followed by subcutaneous inoculation in the flank with 9 × 10^6^ Molm13 or Molm13-XR cells suspended in a 1:1 homogeneous mixture of PBS and Matrigel Matrix (Corning). The tumor volume was calculated as follows: volume (mm^3^) = ½ × (width)^2^ × length. When the tumor size reached 200 mm^3^, mice were randomly divided into three groups (*n* = 8–10 mice/group), and DMSO or pyrvinium (0.5 or 0.8 mg/kg in 20% DMSO in PBS) was administered for six days, followed by a one-day rest. The protocol for the xenograft experiments was approved by the Institutional Animal Care and Use Committee (IACUC) of the College of Medicine, National Taiwan University (no. 20170161, 19 July 2017), and conformed with the criteria outlined in the Guide for the Care and Use of Laboratory Animals prepared by the National Academy of Sciences and published by the National Institutes of Health.

### 2.11. Statistical Analysis

The differences between the measured values were evaluated using a two-sided Student’s t-test. The error bars in the graphs indicate the means ± standard deviation, or means ± standard error of means of at least three independent experiments, conducted in triplicate. Values of *p* < 0.05 were considered statistically significant (* *p* < 0.05; ** *p* < 0.01; *** *p* < 0.001).

## 3. Results

### 3.1. Pyrvinium Pamoate Can Inhibit Cell Proliferation and Induce Cell Death in Molm13 Cells

**Figure 1:**
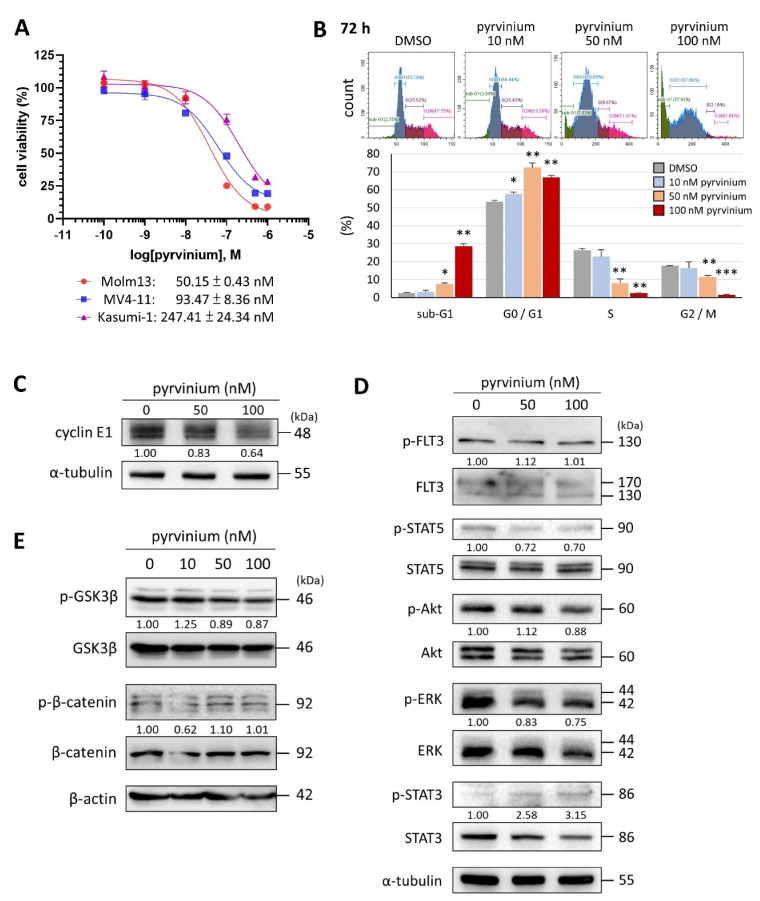
In vitro effect of pyrvinium pamoate on *FLT3*-ITD harboring Molm13 myeloid leukemia cells. (**A**) Half-maximal inhibitory concentration of pyrvinium after 72 h of treatment in Molm13, MV4-11, and Kasumi-1 cells, evaluated by MTS assay. (**B**) After exposure to pyrvinium for 72 h, the different populations of Molm13 cells were analyzed by flow cytometry using propidium iodide staining. * *p* < 0.05, ** *p* < 0.01, *** *p* < 0.001 vs. DMSO. (**C**) Immunoblot analysis of indicated proteins in Molm13 cells treated with DMSO, 50 nM pyrvinium, and 100 nM pyrvinium for 24 h and probed with anti-cyclin E1 antibody. (**D**) Immunoblot analysis of indicated proteins in Molm13 cells exposed to drugs for 4 h; or (**E**) probed with anti-p-GSK3β, GSK3β, p-β-catenin, and β-catenin antibodies. α-Tubulin was used as a loading control. Representative Western blots of three independent experiments are shown. Values represent the fold change with respect to the DMSO control group, after normalization with α-tubulin or β-actin.

To evaluate the efficacy of pyrvinium against leukemia, several myeloid leukemia cell lines were examined. Among them, *FLT3*-ITD-harboring leukemic cells Molm13 were the most sensitive to pyrvinium treatment, exhibiting an IC_50_ of 50.15 ± 0.43 nM (Figure 1A). Flow cytometry analysis showed that pyrvinium triggered cell cycle arrest in the G_0_/G_1_ phase and increased the sub-G_1_ population (Figure 1B). Immunoblotting also revealed the decreased levels of cyclin E1 protein following pyrvinium treatment (Figure 1C). In brief, pyrvinium induced cell cycle arrest and apoptosis-mediated cytotoxicity at doses in the nanomolar range. In this study, we used the percentage of sub-G_1_ populations instead of the percentage of PI/Annexin V (+) populations to evaluate the extent of apoptosis, since the fluorescence background interference of pyrvinium in the PI/Annexin V signals in flow cytometry may have compromised the reliability of the results [34]. The immunoblotting results revealed that FLT3 downstream signaling, including AKT, STAT5, and ERK, in Molm13 cells was only slightly inhibited by pyrvinium (Figure 1D). Further examination showed that pyrvinium did not inhibit other pathways known to be targeted by pyrvinium in other types of tumors, such as the STAT3 and Wnt pathways (Figure 1D,E); these results suggested that alternative mechanisms, including but not limited to mitochondrial mechanisms, may contribute to this robust anti-leukemic activity of pyrvinium.

### 3.2. Transcriptome Analysis Revealed That Pyrvinium Pamoate Triggers an UPR-like Pathway and Redox Balance Modulation, but Blunts Protein Folding Function and ATP Synthesis in Molm13 Cells

**Figure 2:**
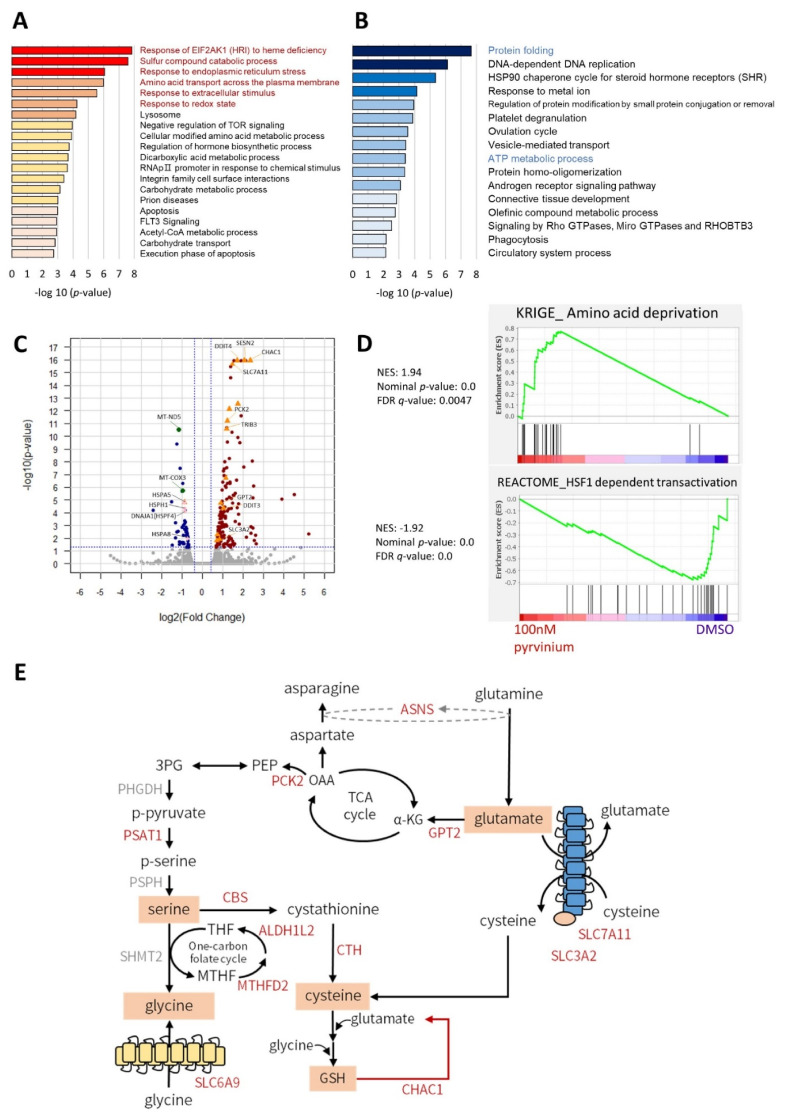
Transcriptomic analysis of 100 nM pyrvinium-treated Molm13 cells. (**A**) Pathway enrichment analysis of upregulated differentially expressed genes (DEGs) in Molm13 cells exposed to 100 nM pyrvinium for 6 h; analysis was performed using Metascape. (**B**) Volcano plot depicting the DEGs upregulated or downregulated by treatment with 100 nM pyrvinium. Orange triangle: ATF4 downstream genes; pink diamond: heat shock protein genes; green dot: mitochondria-encoded genes. (**C**) Gene set enrichment analysist plot of Molm13 cells treated with 100 nM pyrvinium showing the upregulation of genes associated with amino acid deprivation (upper), and the inhibition of HSF1 activation (lower). NES: normalized enrichment score. (**D**) Pathway enrichment analysis of downregulated DEGs in Molm13 cells exposed to 100 nM pyrvinium for 6 h; analysis was performed using Metascape. (**E**) Schematic picture of potential metabolic gene triggered after pyrvinium treatment in Molm13 cells.

To elucidate the alternative mechanisms involved in the action of pyrvinium in Molm13 leukemic cells, we performed whole transcriptome analysis (RNA-seq) on Molm13 cells after treatment with 100 nM pyrvinium for 6 h. A total of 219 genes were identified as significant DEGs between the pyrvinium-treated and the control group, at *p*-value ≤ 0.05. Gene expression was considered to be significantly up- or down-regulated when [log_2_ (fold change)] ≥ 1 or ≤−1, respectively. Among them, 162 upregulated and 57 downregulated genes were observed in the group treated with pyrvinium. Pathway enrichment analysis conducted by Metascape showed that genes upregulated after pyrvinium treatment were strongly associated with ‘response to extracellular stimulus’ [*p*-value = 2.78 × 10^−6^, *q*-value = 0.0079], ‘endoplasmic reticulum stress’ [*p*-value = 8.67 × 10^−7^, *q*-value = 0.0039] or ‘redox balance’ [*p*-value = 5.80 × 10^−5^, *q*-value = 0.0492]; as well as with pathways associated with ‘sulfur compound catabolic process’ [*p*-value = 2.58 × 10^−8^, *q*-value = 0.0003] and ‘amino acid transport’ [*p*-value = 1.02 × 10^−6^, *q*-value = 0.0039] (Figure 2A). A volcano plot was used to depict the genes associated with responses to extracellular stimulus (such as, *SESN2*, and *PCK2*), redox balance (*CTH* and *CHAC1*) and amino acid metabolism (*ASNS*, *GPT2*, *PSAT1*, *SLC7A11*, and *SLC3A2*) (Figure 2C; Supplementary Table S3). GSEA of the transcriptome (Figure 2D, upper) indicated a trend consistent with that of a documented gene signature changed after amino acid deprivation [35].

In contrast, the genes that were differentially downregulated after pyrvinium treatment were enriched in the pathway associated with ‘protein folding modulation’ (response to topologically incorrect protein) [*p*-value = 2.10 × 10^−8^, *q*-value = 0.0005], including several heat shock proteins (HSPs), such as *HSP90B1* (encodes GRP94, *p* = 0.053), *HSPA5* (encodes GRP78), *HSPA8*, *HSPH1*, and *DNAJA1* (HSPF4), and those associated with ‘ATP synthesis’ (ATP metabolism) [*p*-value = 3.95 × 10^−4^, *q*-value = 0.1736], including *MT-ND5* and *MT-COX3* (Figure 2B,C; Supplementary Table S3). GSEA showed that the changes in the transcriptome profile induced by pyrvinium were opposite to the changes in genes after heat shock factor 1 (HSF1) activation (Figure 2D, lower). Based on the results of these transcriptomic analyses, we proposed that pyrvinium could induce a stress response, causing an amino acid deprivation-like scenario, and elicit metabolic rewiring toward amino acid synthesis, transport, and glutathione metabolism (Figure 2E).

### 3.3. Pyrvinium Pamoate Targets Mitochondria and Has an Inhibitory Effect on Mitochondrial Respiration

**Figure 3:**
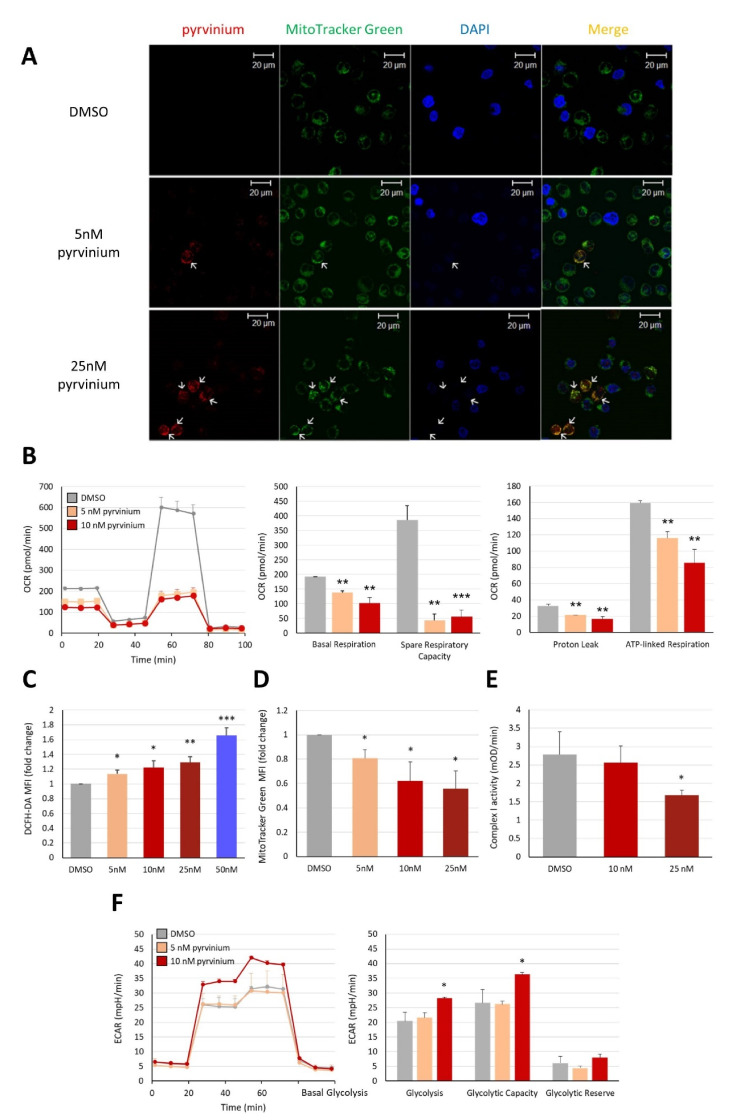
Mitochondria functions were impaired upon exposure to pyrvinium. (**A**) Confocal microscopy images showing that pyrvinium co-localized with mitochondria in Molm13 cells. Arrows indicate the mitochondria overlapped with pyrvinium. (**B**) Seahorse Cell Mito Stress Test was performed to measure basal respiratory, spare respiratory capacity, proton leak, and ATP-linked respiration in Molm13 cells treated with 5 nM, or 10 nM pyrvinium for 24 h compared to those in cells treated with DMSO. (**C**) Intracellular reactive oxygen species levels were measured by DCFH-DA staining; (**D**) Mitochondria mass was determined by MitoTracker Green staining coupled with flow cytometry in Molm13 cells treated with indicated concentrations of pyrvinium for 24 h. (**E**) Mitochondria respiratory chain complex I activity was measured after treatment with DMSO, 10 nM pyrvinium or 25 nM pyrvinium for 24 h. (**F**) Seahorse Glycolysis Stress Test was performed to measure basal glycolysis, glycolytic capacity, and glycolytic reserve after pyrvinium or DMSO treatment for 24 h. * *p* < 0.05, ** *p* < 0.01, *** *p* < 0.001 vs. DMSO.

Pyrvinium has been reported to co-localize with mitochondria and cause mitochondrial stress, leading to the inhibition of complex I activity, impediment of mitochondrial oxidative phosphorylation (OXPHOS), and a decreased ATP production in human myeloma and erythroleukemia cell lines [12]. To clarify whether treatment with pyrvinium could alter the mitochondria function in Molm13 leukemia cells, the cellular location of pyrvinium, electron transport chain enzyme activity (especially complex I), and ATP production were examined. MitoTracker Green staining, coupled with confocal microscopy, showed that the red fluorescence of pyrvinium overlapped with the green fluorescence of the mitochondria, indicating that pyrvinium was localized in the mitochondria (Figure 3A). Subsequently, Seahorse bioanalyzer analysis revealed that pyrvinium had an inhibitory effect on the mitochondrial basal respiration rate, spare respiratory capacity, proton leak, and ATP production after treatment with 10 nM pyrvinium for 24 h (Figure 3B). Further evaluation of the ROS levels and total mitochondrial mass was conducted by DCFH-DA and MitoTracker Green staining, respectively. This showed that pyrvinium increased ROS levels (Figure 3C), but reduced the mass of mitochondria (Figure 3D). Finally, the dose-dependent inhibition of the mitochondrial complex I was noted (Figure 3E). On the other hand, to evaluate the effect of pyrvinium on glycolytic activity in Molm 13 cells, the Glycolysis Stress Test was performed, revealing that basal glycolysis and glycolytic capacity were increased after pyrvinium treatment (Figure 3F). In conclusion, pyrvinium could trigger mitochondrial stress by increasing ROS levels and impede mitochondrial functions, as indicated by the decreased OCR, ATP production, mitochondrial mass, and complex I activity.

### 3.4. Pyrvinium Pamoate Triggers the Activation of the eIF2α-ATF4 Pathway and Inhibits mTORC1 Signaling

**Figure 4:**
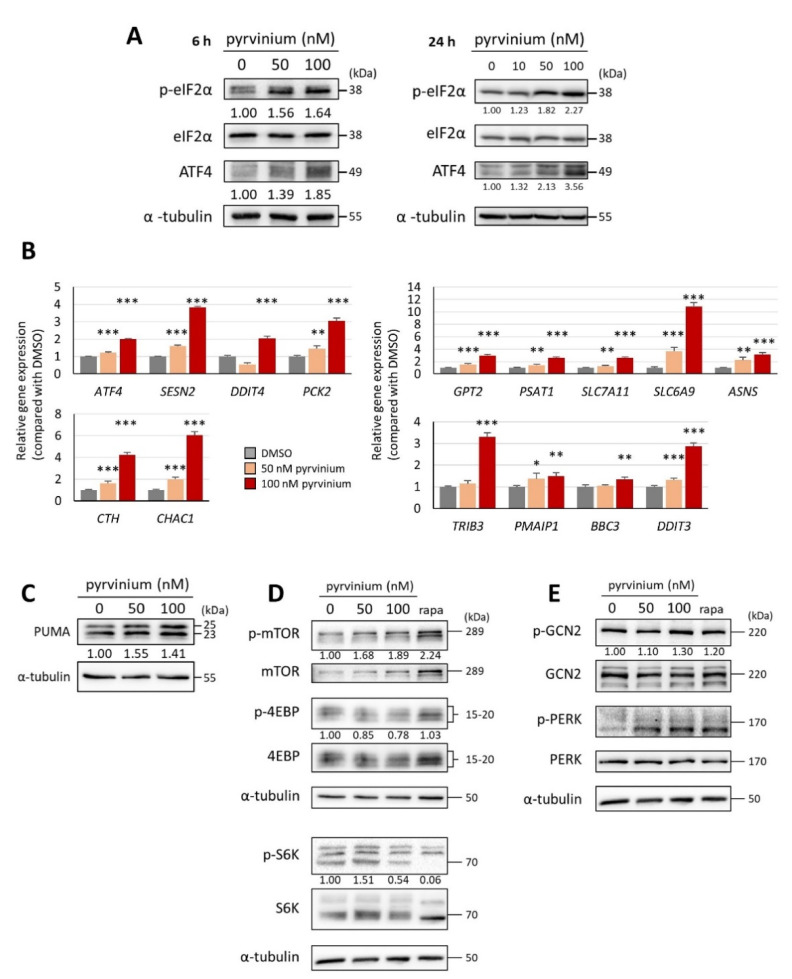
Pyrvinium modulated signaling pathways associated with the integrated stress response in Molm13 cells. (**A**) Western blot analysis of Molm13 cells treated with DMSO, 50 nM pyrvinium, or 100 nM pyrvinium, then probed with antibodies against p-eIF2α, eIF2α, ATF4, and α-tubulin (loading control) for 6 h (left) and 24 h (right). (**B**) q-RT-PCR analyses of indicated genes in Molm13 cells upon exposure to DMSO, 50 nM pyrvinium, or 100 nM pyrvinium for 24 h. * *p* < 0.05, ** *p* < 0.01, *** *p* < 0.001 vs. DMSO. Western blot analysis of Molm13 treated with DMSO, 50 nM pyrvinium, 100 nM pyrvinium, or 20 nM rapamycin (positive control), then probed with antibodies against (**C**) PUMA, (**D**) p-mTOR, mTOR, p-4EBP, 4EBP, p-S6K, or S6K, (**E**) p-GCN2, GCN2, p-PERK, or PERK, and α-tubulin (loading control) for 24 h. Representative Western blots of three independent experiments are shown. Values represent the fold change with respect to the DMSO control group after normalization with α-tubulin.

To elucidate the role of integrated stress response (ISR) in the activity of pyrvinium, the phosphorylation of eukaryotic initiation factor 2α (eIF2α) and the expression of activating transcription factor 4 (ATF4) were examined. A dose-dependent and time-dependent increase in the total ATF4 and phosphorylated elF2α levels was noted following pyrvinium treatment (Figure 4A). In addition, the expression of various ATF4-regulated genes, which are associated with stress response, redox balance, and amino acid synthesis or transport, was also increased in a dose-dependent manner; this was confirmed by q-RT-PCR (Figure 4B). These findings indicated that pyrvinium may elicit a cellular stress response via the eIF2α-ATF4 signaling pathway. Notably, the gene expression of *TRIB3* (involved in C/EBP homologous protein-10 [CHOP]-related cell death) [36], *PMAIP1* (encoding proapoptotic NOXA), *BBC3* (encoding proapoptotic p53 upregulated modulator of apoptosis [PUMA]), and *DDIT3* (CHOP) was also stimulated after 24 h of pyrvinium treatment (Figure 4B); this is in line with the pyrvinium-induced apoptosis which was observed in Figure 1B. Upregulation of the protein level of PUMA was also observed after exposure to pyrvinium for 24 h (Figure 4C). In addition, increased gene expression of DNA damage inducible transcript 4 (*DDIT4*) and sestrin 2 (*SESN2*), two ATF4-regulated mTORC1 inhibitors, was noted following pyrvinium exposure (Figure 4B). Western blotting also revealed that the phosphorylation of the mTORC1 downstream proteins, ribosomal protein S6 kinase 1 (S6K) and eukaryotic translation initiation factor 4E-binding protein 1 (4E-BP1), was reduced after 24 h treatment with pyrvinium (Figure 4D); however, the phosphorylation of the protein kinase R-like endoplasmic reticulum kinase (PERK) and general control nonderepressible 2 (GCN2) was not obviously increased (Figure 4E). Therefore, we suggested that pyrvinium could trigger the activation of the eIF2α-ATF4 pathway, in a PERK- and GCN2-independent manner, to promote the expression of its downstream effectors, DDIT4 and SESN2, and thus inhibit the mTORC1 signaling pathway. This is in line with the report that the eIF2α-ATF4 pathway could be activated through mitochondrial stress, without classical kinase activation [37].

### 3.5. Pyrvinium Pamoate Exhibited Similar Activity in Molm13 Cells and Cabozantinib-Resistant Molm13-XR Cells

Cabozantinib is a tyrosine kinase inhibitor (TKI) that exhibits substantial activity against specific myeloid leukemia cell lines with *FLT3*-internal tandem duplication (ITD) [38] or *AML1-ETO*/*KIT*mt [29] mutations, including MV4-11, Molm13, and Kasumi-1 cells. It is known that most patients with AML carrying FLT3-ITD show an initial favorable response to TKIs, such as midostaurin and gilteritinib; however, the emergence of drug-resistance can decrease drug efficacy [4]. To investigate whether pyrvinium can act on TKI-resistant cell lines, we established a cabozantinib-resistant Molm13 cell line (Molm13-XR) by exposing Molm13 cells to gradually increasing concentrations of cabozantinib. The IC_50_ of cabozantinib increased from 1.06 ± 0.93 nM in Molm13 cells to 473.36 ± 154.73 nM in Molm13-XR cells.

**Figure 5:**
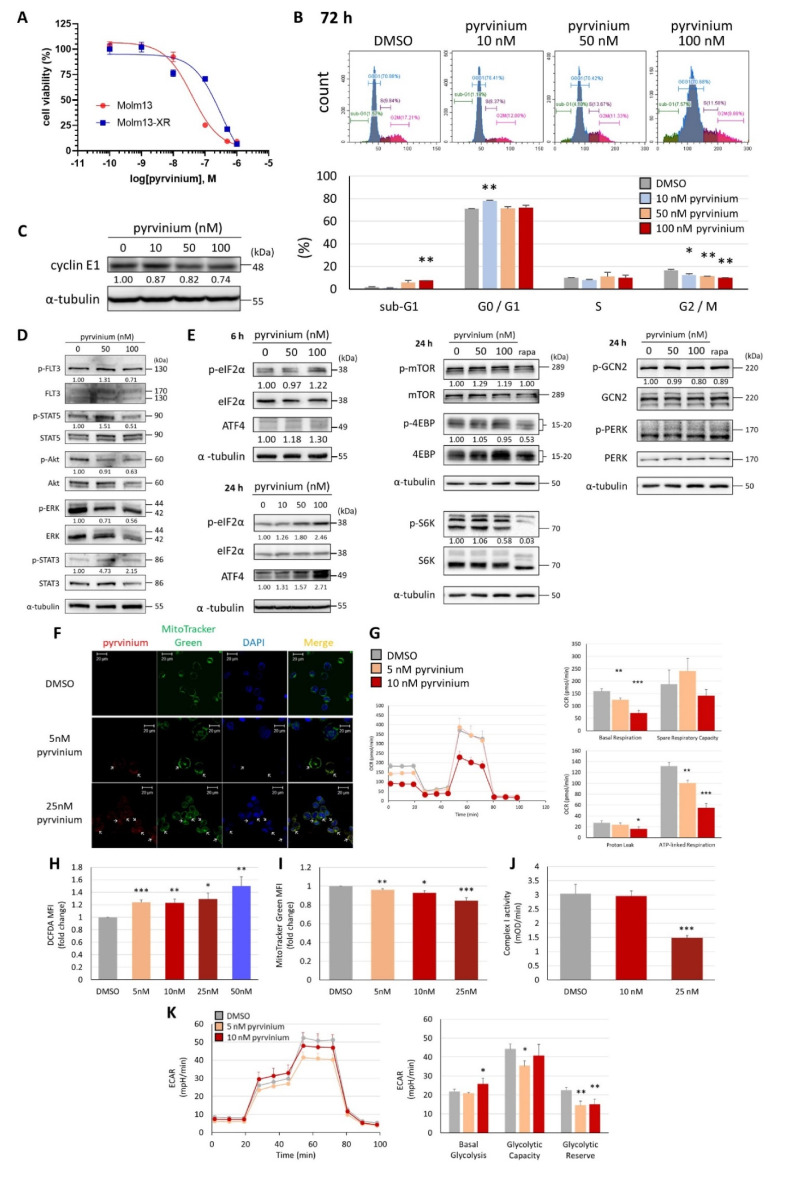
In vitro anti-leukemic effect of pyrvinium in Molm13-XR cells. (**A**) Half maximal inhibitory concentration of pyrvinium after treatment for 72 h, evaluated by the MTS assay in Molm13, and Molm13-XR cells. (**B**) Different populations of Molm13-XR cells upon exposure to pyrvinium for 72 h were evaluated by flow cytometry using propidium iodide staining. * *p* < 0.05, ** *p* < 0.01, *** *p* < 0.001 vs. DMSO. (**C**) Western blot analysis of Molm13-XR cells treated with DMSO, 50 nM pyrvinium, or 100 nM pyrvinium for 24 h and probed with an anti-cyclin E1 antibody. (**D**) Western blot analysis of indicated proteins in Molm13-XR cells exposed to drugs for 4 h. (**E**) Western blot analysis of Molm13-XR cell lysates probed with anti-p-eIF2α, eIF2α, and ATF4 antibodies for 6 h (**E**, upper left) or 24 h (**E**, lower left); or probed with anti-p-mTOR, mTOR, p-4EBP, 4EBP, p-S6K, S6K, p-GCN2, GCN2, p-PERK, PERK antibodies for 24 h (**E**, middle and right). α-Tubulin was used as a loading control. Representative Western blots of three independent experiments are shown. Values represent the fold change with respect to the DMSO control group after normalization with α-tubulin. (**F**) Confocal microscopy images demonstrating that pyrvinium was co-localized with mitochondria in Molm13-XR cells. Arrows indicate the mitochondria overlapped with pyrvinium. (**G**) Seahorse Cell Mito Stress Test was performed to measure basal respiratory, spare respiratory capacity, proton leak, and the ATP-linked respiratory in Molm13-XR cells treated with 5 nM, or 10 nM pyrvinium for 24 h, compared with those in cells treated with DMSO. (**H**) Intracellular reactive oxygen species levels were measured by DCFH-DA staining; (**I**) Mitochondria mass was determined by MitoTracker Green staining coupled with flow cytometry in Molm13-XR cells treated with indicated concentrations of pyrvinium for 24 h. (**J**) Mitochondria respiratory chain complex I activity was measured after treatment with DMSO, 10 nM pyrvinium or 25 nM pyrvinium for 24 h. (**K**) Seahorse Glycolysis Stress Test was performed to measure basal glycolysis, glycolytic capacity, and glycolytic reserve after pyrvinium or DMSO treatment for 24 h. * *p* < 0.05, ** *p* < 0.01, *** *p* < 0.001 vs. DMSO.

We subsequently investigated the efficacy of pyrvinium against Molm13-XR cells. Similar to the results in Molm13 cells, pyrvinium also inhibited the proliferation of Molm13-XR in a dose-dependent manner, exhibiting an IC_50_ of 115.5 ± 23.04 nM (Figure 5A). Pyrvinium also induced apoptosis (Figure 5B), decreased the population of cells in the G_2_/M phase, and decreased the cyclin E1 protein level in Molm13-XR cells after treatment for 72 h (Figure 5C). Additionally, pyrvinium also inhibited the AKT, STAT5, and ERK signaling pathways (Figure 5D). Pyrvinium also triggered eIF2α activation, increased ATF4 protein levels, and inhibited the mTOR signaling pathway (Figure 5E). Pyrvinium could co-localize with mitochondria (Figure 5F) and further reduce the mitochondria basal respiratory rate and mitochondrial ATP production (Figure 5G) in the cabozantinib-resistant cell line. Similarly, pyrvinium could increase ROS levels, decrease mitochondrial mass, and inhibit mitochondria complex I activity (Figure 5H–J) in Molm13-XR cells. On the other hand, the Glycolysis Stress Test revealed that only basal glycolysis was increased but glycolytic capacity was decreased after pyrvinium treatment (Figure 5K).

### 3.6. Pyrvinium Pamoate Retards the Growth of Subcutaneous Molm13 and Molm13-XR Xenograft Tumors

**Figure 6:**
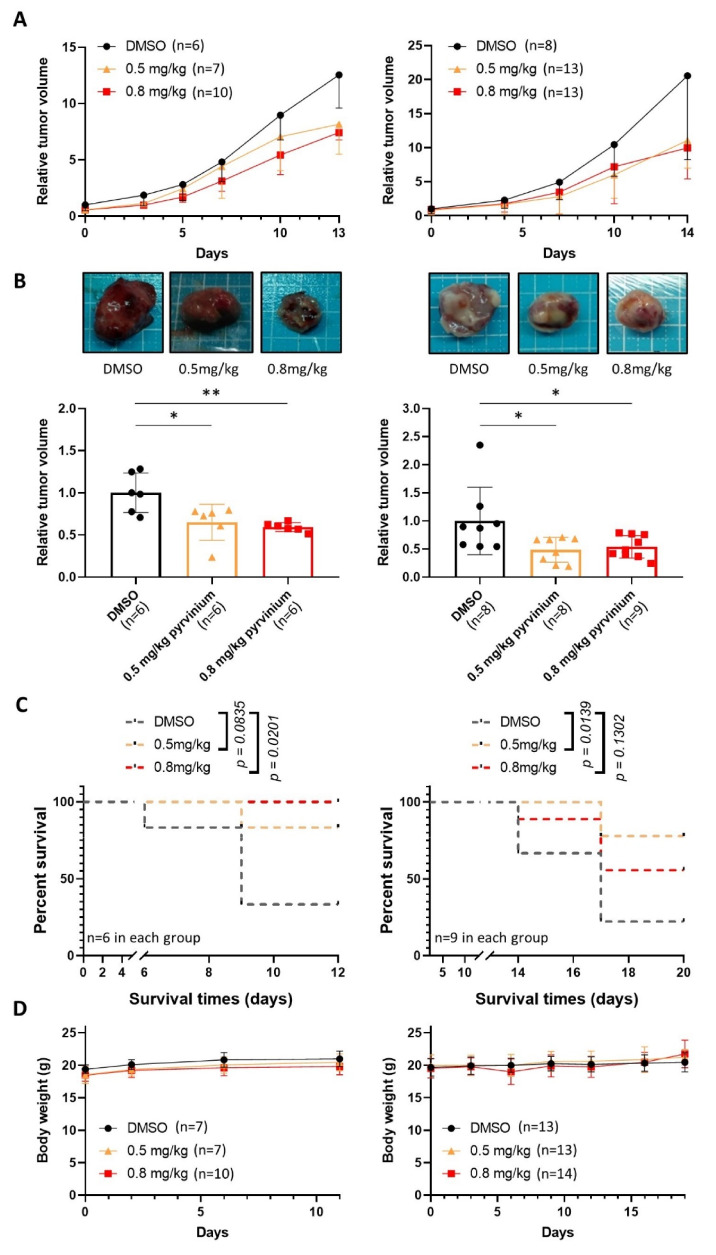
Pyrvinium pamoate impedes the growth of subcutaneous Molm13 and Molm13-XR xenograft tumors in mice. (**A**) The relative tumor volume of the Molm13 (left) or Molm13-XR (right) subcutaneous xenografts was recorded every 2 or 3 days when DMSO, 0.5 mg/kg pyrvinium, or 0.8 mg/kg pyrvinium was administrated by intraperitoneal injection as a function of time in the three groups. (**B**) Comparison of tumor volume of the Molm13 (left) and Molm13-XR (right) tumors isolated from each group 13 and 14 days, respectively, after treatments. (**C**) Kaplan–Meier survival curves of different groups of mice bearing Molm13 (left) and Molm13-XR (right) were plotted against days after treatment with pyrvinium or DMSO. (**D**) The body weight of mice bearing Molm13 (left) or Molm13-XR (right). Data represent the mean ± SD for each group. * *p* < 0.05; ** *p* < 0.01. n.s., statistically non-significant.

To determine the antitumor efficiency of pyrvinium in vivo, mice bearing Molm13 and Molm13-XR tumors were injected intraperitoneally with pyrvinium (0.5 or 0.8 mg/kg), six days a week. We found that the pyrvinium treatment retarded Molm13 (Figure 6A,B, left) and Molm13-XR (Figure 6A,B, right) tumor growth in a dose-dependent manner. Compared with the control group (DMSO), intraperitoneal administration of pyrvinium at a dose of 0.8 mg/kg could significantly prolong the survival time of mice inoculated with Molm13 and Molm13-XR tumors (Figure 6C). The drug was well-tolerated in mice and no effects on the body weight were observed during the dosing period (Figure 6D).

## 4. Discussion

In this study, we revealed that pyrvinium pamoate could not only inhibit the FLT3 signaling pathway, but also target mitochondria and induce cellular stress responses; these ultimately inhibited cell proliferation and triggered the apoptosis of Molm13 and derived Molm13 (Molm13-XR) myeloid leukemia cells (Supplementary Figure S1). To the best of our knowledge, this is the first study to comprehensively report the anti-malignant effect of pyrvinium in acute myeloid leukemia cells through transcriptomic analysis and extensive functional assays.

Several anti-cancer mechanisms of pyrvinium have been reported in solid tumors, including the regulation of multiple signaling pathways (such as β-catenin, AKT, STAT3, and Hedgehog signaling) which support cell proliferation and survival, and the regulation of cellular response signaling (such as autophagy and UPR), which is observed in several solid tumor malignancies [13]; however, reports of the role of pyrvinium in myeloid leukemia are limited [12,39]. Harada et al. reported that pyrvinium could inhibit ATP production, STAT3 phosphorylation, and human erythroleukemia (HEL 92.1.7) cell proliferation, but did not have this activity in corresponding cells without mitochondrial DNA [12]. Another report focused on two acute monocytic leukemia cell lines, SHI-1 and THP-1, both of which carry the mixed-lineage leukemia (MLL)-rearrangement. They found that pyrvinium co-localized with mitochondria in cells and concluded that the drug could inhibit mitochondrial respiration and was highly effective against MLL-rearranged AML cells in vitro [39]. In this study, we evaluated the effect of pyrvinium on mitochondrial functions from various aspects and highlighted ISR as an alternative downstream message of mitochondrial stress that exerts a cytotoxicity effect partly in an STAT5-associated manner.

Our whole transcriptome analysis further revealed that pyrvinium could induce obvious ISR, as demonstrated by the activation of the eIF2α -ATF4 pathway, and the subsequent increase in the transcription of the downstream genes associated with redox balance (CTH and CHAC1), amino acid synthesis and transport (SLC7A11, SLC6A9, ASNS), and metabolic reprogramming (GPT2, PCK2, PSAT1). Normal ISR signaling could primarily act through a protective mechanism; however, prolonged ISR would promote cell death through induction of the expression of a series of pro-apoptotic genes, including BBC3 (encodes PUMA) and PMAIP1 (encodes NOXA). It is worth noting that after pyrvinium treatment, the expression of DDIT3, TRIB3 and CHAC1 genes, which were reported to be associated with CHOP-related cell death [36,40,41], was stimulated, contributing to pyrvinium-induced apoptosis. In addition, ISR was reported to play a crucial role in hematopoietic stem cells and contribute to their persistence [42], indicating the importance of the optimal regulation of the ISR for the survival of leukemic cells. Other series studies investigating ONC201 and ONC212 (imipridone) suggested that the upregulation of ISR could promote anti-cancer activity against solid and hematological malignancies [43,44,45,46], and help overcome the resistance to the B-cell lymphoma (BCL)-2 inhibitor venetoclax [47]. Although ONC201 and ONC212 exhibit similar effects on the ISR, pyrvinium, being an FDA-approved drug, may be more suitable for further clinical trials as part of drug repurposing for acute myeloid leukemia.

The pathway enrichment analysis by Metascape also revealed that exposure of cells to pyrvinium caused the downregulation of the ATP metabolic process and a decrease in the expression of several genes involved in the mitochondria electron transport chain, including MT-COX3 (cytochrome c oxidase subunit III), MT-CYB (cytochrome b), and MT-ND5 (subunit of complex I). To determine whether pyrvinium-induced ISR is associated with mitochondria, we further studied the effect of pyrvinium on the mitochondria complex and its function. In this study, we demonstrated that pyrvinium co-localized with mitochondria and caused mitochondrial stress, presented by the inhibition of complex I activity, impediment of mitochondrial OXPHOS, and a decrease in both mitochondrial mass and ATP production. Likewise, an antimalarial compound atovaquone has also been reported to simultaneously induce ISR and suppress OXPHOS [48]. Recently, Hlozkova and others detected the basal metabolic profile of several leukemia cell lines, including Kasumi-1, Molm13, and MV4-11 cells [49]. Among them, Kasumi-1 cells had the highest mitochondrial function, with the highest basal respiration, ATP-linked respiration, and maximal respiration, while Molm13 and MV4-11 cells had similar mitochondrial function. However, in our study the IC_50_ of pyrvinium pamoate in Kasumi-1 cells was higher than that in Molm13 and MV4-11 cells (Figure 1A). In addition, our unpublished results showed that the maximal respiration of the Molm13-XR cells was significantly higher than that of Molm13 cells, and its basal respiration and ATP-linked respiration also tended to be higher than that of Molm13 cells. This information indicates that we could not conclude that cells with a higher mitochondrial function are more susceptible to pyrvinium pamoate. We also evaluated ECAR using the glycolysis stress test, which demonstrated increased basal glycolysis and glycolytic capacity after treatment with 10 nM pyrvinium in Molm13 cells. However, the compensation for the upregulated glycolysis rate via mitochondria OXPHOS reduction could not rescue the loss of the total ATP. In contrast, the compensatory increase in glycolysis was not observed in Molm13-XR cells after pyrvinium treatment, which may be due to the cell’s original higher glycolytic capacity than that of its parental cell line. In addition, we observed that pyrvinium could cause the accumulation of ROS in Molm13 cells, and it has been reported to selectively trigger the ROS release and mitochondrial membrane depolarization, as well as inhibit aerobic glycolysis in KRAS-mutant lung cancer cells [50]. Low doses of ROS can play an important role in cell cycle progression, proliferation, differentiation, and cell migration [51], while higher doses can contribute to ISR activation [52]. This information supports our hypothesis regarding the connection between mitochondria dysfunction and the integrated stress response triggered by pyrvinium.

GSEA of our transcriptomic profile revealed that a set of genes, which are upregulated after amino acid deprivation, were also upregulated after pyrvinium treatment, indicating that pyrvinium treatment could trigger a cell response similar to that of amino acid deprivation (Figure 2C, upper). Further, cancer cells exhibit higher demand for amino acids [53] and have better responses to amino acid-deprivation therapies targeting amino acid uptake and catabolism than normal cells [54]. Furthermore, hematological malignancies frequently develop dependency on specific amino acids for their survival, and insufficient production of these amino acids may induce a metabolic vulnerability and therapeutic opportunities [55]. For example, glutaminolysis was identified as synthetically lethal with FLT3-TKI treatment, indicating the potency of using amino acid depletion in combination therapies [56,57]. Another FLT3-TKI gilteritinib was also proved to hinder glutamine uptake and utilization in *FLT3*-ITD–positive AML [58]. Recently, NEI-01, a novel arginine-depleting enzyme, could not only induce arginine deprivation, but also had cytotoxic activity against arginine auxotrophic AML cells through induction of cell cycle arrest and apoptosis [59]. Moreover, ADI-PEG20, which degrades arginine to citrulline, could prevent the proliferation of tumor cells in several cancers [60], and a further study showed its efficacy when combined with cytarabine in treating AML in mouse models [61]. A phase I study of ADI-PEG20 plus low-dose cytarabine for the treatment of AML is ongoing [62]. Given that pyrvinium pamoate induces a similar transcriptomic pattern to amino acid deprivation, it is important to determine the amino acids whose synthesis is disrupted by pyrvinium pamoate to identify alternative metabolic vulnerabilities to develop a combination strategy with chemotherapy.

Furthermore, to explore the mechanism by which pyrvinium induces the inhibition of Molm13 cells, several chemicals affecting gene expression patterns in a similar manner as pyrvinium were screened using a connectivity map [63,64]. Interestingly, androgen receptor inhibitors (idazoxan, phenoxybenzamine) [23], K^+^ ionophores (valinomycin) [65], mTOR inhibitors (sirolimus), and PI3K inhibitors (LY-294002) were found to exhibit similar effects on genome-wide gene expression as those of pyrvinium (Supplementary Figure S2; Table S4).

We demonstrated the downregulation of the protein folding pathway, which includes several HSP genes, such as HSP90B1, HSPA, HSPA8, HSPH1, and DNAJA1 (HSPF4). Activation of HSF1, which transcriptionally triggers the mRNA expression of HSPs [66], was significantly inhibited by pyrvinium, as demonstrated by GSEA (Figure 2C, lower). Previous research suggested that HSF1 activation would be inhibited by amino acid starvation [67] or mTOR inhibition [68]. Finally, we discovered that pyrvinium could induce apoptosis in both Molm13 and Molm13-XR cells (a cabozantinib-resistant Molm13 cell line), indicating that targeting mitochondrial respiration and ISRs might not only be effective in the treatment of AML, but also in overcoming cabozantinib resistance. Further transcriptome comparison indicated a little overlap between cabozantinib-resistant DEGs and pyrvinium-induced DEGs (Supplementary Figure S3), which may be the reason why Molm13-XR cells were still sensitive to pyrvinium.

## 5. Conclusions

In summary, this study provides an example of a drug repurposing strategy. We found that pyrvinium could localize to mitochondria, block complex I activity, elevate ROS levels, and then trigger a pro-apoptotic ISR in both *FLT3*-ITD-positive Molm13 cells and cabozantinib-resistant Molm13-XR cells. These findings suggest that pyrvinium can be used in the treatment of AML in the future.

## Data Availability

The transcriptome data presented in this study are available in the Gene Expression Omnibus (GEO) database (ID: GSE153854).

## Supplementary Materials

The following are available online at https://www.mdpi.com/article/10.3390/biomedicines9121869/s1, Figure S1: Schematic overview of the mechanisms underlying the anti-leukemic activity of pyrvinium in Molm13 myeloid leukemia cells, Figure S2: Chemical screening by connectivity map (build02) to identify molecules influencing gene expression in a similar manner to pyrvinium treated Molm13 cells, Figure S3: Comparison of DEGs between transcriptomics from pyrvinium-treated Molm13 cells (pyrvinium) and those from cabozantinib-resistant (XR) cells. Table S1: Primary antibodies used in this research, Table S2: Primer sequences for quantitative reverse transcription-PCR, Table S3: List of upregulated and downregulated genes after pyrvinium treatment, Table S4: Candidate list of molecules from connectivity map analysis.
