# Supplementary material for: Deciphering the Role of Pyrvinium Pamoate in the Generation of Integrated Stress Response and Modulation of Mitochondrial Function in Myeloid Leukemia Cells through Transcriptome Analysis
Source: Biomedicines. 2021 Dec 9;9(12):1869. doi: 10.3390/biomedicines9121869 (PMC8698814; doi:10.3390/biomedicines9121869)
Supplement: Supplementary file 1 [file biomedicines-09-01869-s001.zip › biomedicines-1449184-supplementary.pdf]

Supplementary Information

Supplementary Figure

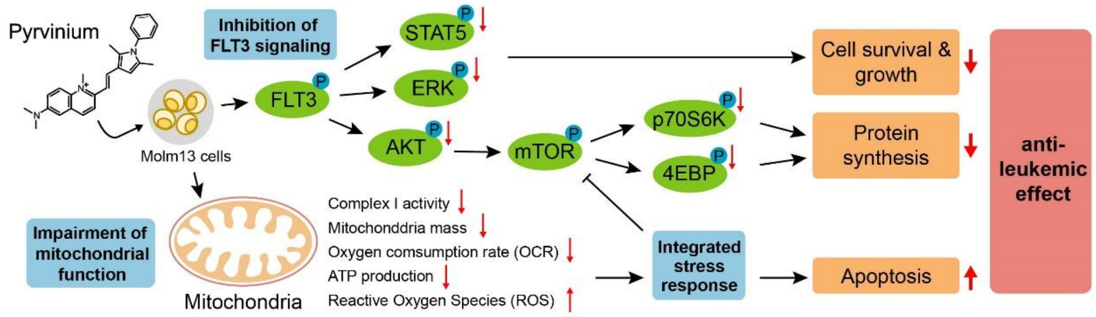

Supplementary Figure S1

Schematic overview of the mechanisms underlying the anti-leukemic activity of pyrvinium in Molm13 myeloid leukemia cells.

Pyrvinium inhibits FLT3 downstream signaling pathways, including the AKT, ERK, and STAT5 signaling pathways. In addition, pyrvinium targets mitochondria and inhibits mitochondrial energy functions, thus inducing cellular stress responses. These effects finally inhibit cell proliferation and induce apoptosis.

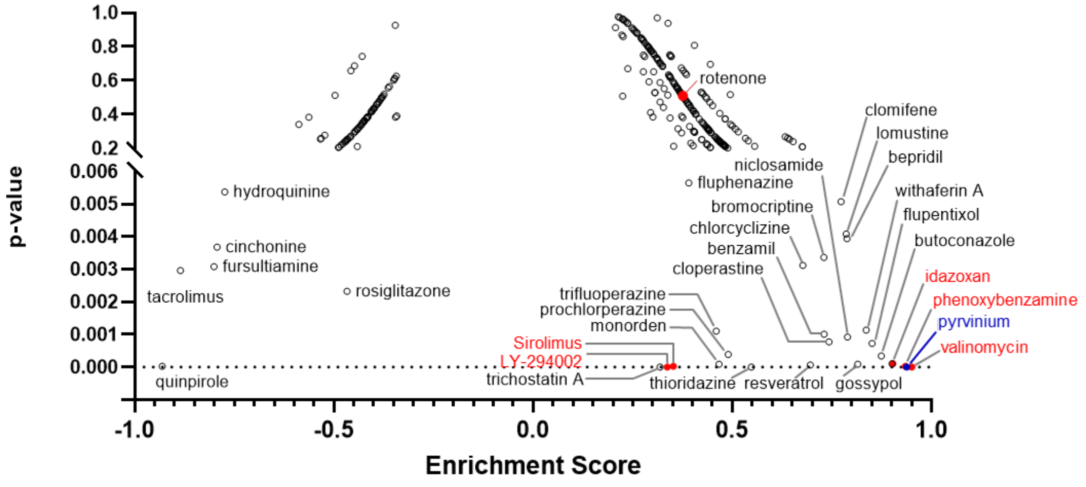

Supplementary Figure S2

Chemical screening by connectivity map (build02) to identify molecules influencing gene expression in a similar manner to pyrvinium treated Molm13 cells.

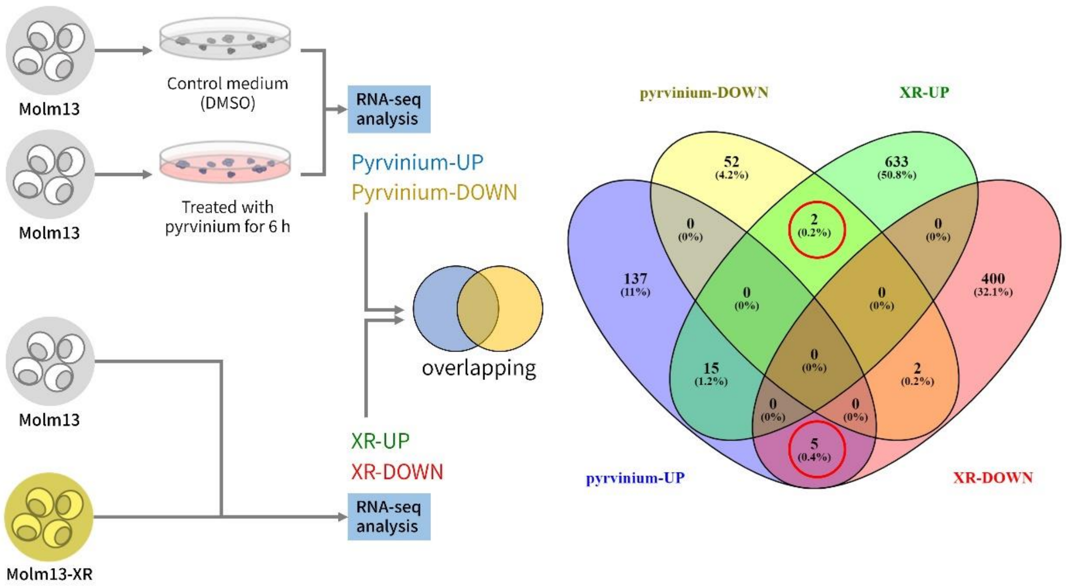

Supplementary Figure S3

Comparison of DEGs between transcriptomics from pyrvinium-treated Molm13 cells (pyrvinium) and those from cabozantinib-resistant (XR) cells by Venny 2.1 (https://bioinfogp.cnb.csic.es/tools/venny/index.html) [1].

Supplementary Table

Supplementary Table S1

Primary antibodies used in this research.

Product	Brand	Catalog no. #	Source
Phospho-FLT3 (Tyr589/Tyr591)	Cell Signaling	#3464	Rabbit
FLT3	Santa Cruz	Sc-479	Mouse
Phospho-mTOR (Ser2448)	Cell Signaling	#2971	Rabbit
mTOR	Cell Signaling	#2972	Rabbit
Phospho-STAT3 (Tyr705)	Cell Signaling	#9131	Rabbit
STAT3	Cell Signaling	#12640	Rabbit
Phospho-STAT5 (Tyr694)	Genetex	GTX61079	Rabbit
STAT5	Genetex	GTX61098	Rabbit
Phospho-AKT(Ser473)	Cell Signaling	#4058	Rabbit
AKT	Cell Signaling	#9272	Rabbit
Phospho-p44/42 ERK 1/2 (Thr202/Tyr204)	Cell Signaling	#4370	Rabbit
p44/42 ERK1/2	Cell Signaling	#4695	Rabbit
α-tubulin	Genetex	GTX628802	Mouse
β-actin	Genetex	GTX109639	Rabbit
Cyclin E1	Cell Signaling	#4129	Mouse
PUMA	Cell Signaling	#4976	Rabbit
Phospho-p70 S6K (Thr389)	Cell signaling	#9205	Rabbit
p70 S6 Kinase	Cell Signaling	#2708	Rabbit
Phospho-4E-BP1 (Thr37/46)	Cell Signaling	#2855	Rabbit
4E-BP1	Cell Signaling	#9644	Rabbit
Phospho-GSK-3-beta (Ser9)	Cell signaling	#9322	Rabbit
GSK-3β	Cell signaling	#9315	Rabbit
Phospho-PERK (T980)	Cell signaling	#3179	Rabbit
PERK	Proteintech	20582-1-AP	Rabbit
Phospho-eIF2α (Ser51)	ABclonal	AP0635	Rabbit
eIF2α (EIF2S1/EIF2A)	Proteintech	11170-1-AP	Rabbit
ATF4	Proteintech	10835-1-AP	Rabbit
Phospho-GCN2 (T899)	CUSABIO	CSB-PA010224	Rabbit
GCN2	CUSABIO	CSB-PA868396ESR2HU	Rabbit

Supplementary Table S2

Primer sequences of quantitative reverse transcription-PCR

Gene	Forward primer (5’-3’)	Reverse primer (5’-3’)
**Cell responses to stress stimulus**		
ATF4	TGGCTGGCTGTGGATGG	TCCCGGAGAAGGCATCCT
SESN2	CCTCTGGGCGAGTAGACAAC	GGAGCCTACCAGGTAAGAACA
PCK2	GCCATCATGCCGTAGCATC	AGCCTCAGTTCCATCACAGAT
DDIT4	TGAGGATGAACACTTGTGTGC	CCAACTGGCTAGGCATCAGC
**Redox balance**		
CTH	CATGAGTTGGTGAAGCGTCAG	AGCTCTCGGCCAGAGTAAATA
CHAC1	GAACCCTGGTTACCTGGGC	CGCAGCAAGTATTCAAGGTTGT
**Amino acid metabolism**		
ASNS	GGAAGACAGCCCCGATTTACT	AGCACGAACTGTTGTAATGTCA
GPT2	GTGATGGCACTATGCACCTAC	TTCACGGATGCAGTTGACACC
PSAT1	TGCCGCACTCAGTGTTGTTAG	GCAATTCCCGCACAAGATTCT
SLC7A11	GCGTGGGCATGTCTCTGAC	GCTGGTAATGGACCAAAGACTTC
SLC6A9	CAGATCGAGTTTGTACTGACGAG	GCGATAGCAGAGGTATGGGAAG
**One carbon cycle**		
ALDH1L2	TAGTCCAAAGCACGGCTCTAT	GGTCCTGTATCCAAGCCATCA
**Protein folding modulation**		
HSP90B1	CCAGTTTGGTGTCGGTTTCTAT	CTGGGTATCGTTGTTGTGTTTTG
HSPA5	GAAAGAAGGTTACCCATGCAGT	CAGGCCATAAGCAATAGCAGC
HSPH1	CCGGAAAGATGAACAGGTCAC	GTGTAGCGCCTCCAACAATC
DNAJA1	ACTGGAGCCAGGCGATATTAT	CTTCAACGAGCTGTATGTCCAT
**ATP synthesis**		
MT-*ND5*	ATCGGTTTCATCCTCGCCTT	AGTCAGGGGTGGAGACCTAA
MT-*COX3*	ACCAATGATGGCGCGATGTA	GGCTGGAGTGGTAAAAGGCT
**Apoptosis**		
DDIT3	AAGGCACTGAGCGTATCATGT	TGAAGATACACTTCCTTCTTGAACA
BBC3	GACCTCAACGCACAGTACGAG	AGGAGTCCCATGATGAGATTGT
PMAIP1	CTGGAAGTCGAGTGTGCTACT	TCAGGTTCCTGAGCAGAAGAG
TRIB3	AAGCGGTTGGAGTTGGATGAC	CACGATCTGGAGCAGTAGGTG
**Internal control**		
18S rRNA	GTAACCCGTTGAACCCCATT	CCATCCAATCGGTAGTAGCG

Supplementary Table S3

List of upregulated and downregulated genes after 100 nM pyrvinium treatment for 6 h

Name	Gene symbol	*p*-value*	Fold change	pyrvinium -treated**	Control-DMSO**
**Upregulated DEGs**					
**Cell responses to stress stimulus**					
	SESN2	0	3.27	1998.65	610.01
	PCK2	6.18E-12	2.32	4914.99	2119.79
**Redox balance**					
	CTH	0	3.68	903.92	245.02
	CHAC1	0	4.18	375.73	89.47
**Amino acid metabolism**					
	ASNS	2.22E-16	2.62	5756.94	2198.07
	GPT2	1.79E-05	1.86	3726.82	2003.89
	PSAT1	5.08E-08	2.03	19022.60	9367.74
	SLC7A1	0.006459	1.66	11107.67	6686.74
	SLC3A2	0.015841	1.63	10313.91	6327.85
**downregulated DEGs**					
**Protein folding modulation**					
	HSP90B1	0.053825	0.63	16575.44	26465.33
	HSPA5	1.54E-05	0.54	9924.41	18403.02
	HSPA8	0.010537	0.60	67141.73	112471.82
	HSPH1	4.68E-05	0.55	11466.68	20886.78
	DNAJA1 (HSPF4)	7.37E-05	0.55	7144.79	12887.50
**ATP synthesis**					
	MT-*ND5*	2.83E-11	0.44	34307.57	77322.98
	MT-*COX3*	1.77E-06	0.51	89840.98	174974.58

* “PPEE” were used as corrected p-values

** The value of “Cabozantinib-treated” and the” Control-DMSO” were obtained from the output of EBseq to calculate the differentially expression (DE), and represented the mean of each transcript within each condition (adjusted by normalization factors).

Supplementary Table S4

Candidate list of molecules from connectivity map analysis

compound name	Enrichment score	P-value	specificity	percent non-null	mean	n
valinomycin	0.951	0	0.0116	100	0.674	4
pyrvinium	0.938	0	0.0046	100	0.727	6
phenoxybenzamine	0.935	0.00002	0.1386	100	0.596	4
idazoxan	0.903	0.0001	0	100	0.551	4
phenazopyridine	0.901	0.0001	0.0065	100	0.646	4
butoconazole	0.874	0.00034	0	100	0.569	4
flupentixol	0.851	0.00072	0	100	0.588	4
withaferin A	0.836	0.00113	0.1	100	0.602	4
gossypol	0.815	0.00008	0	83	0.625	6
niclosamide	0.789	0.00092	0.0105	100	0.496	5
bepridil	0.788	0.00394	0.0444	100	0.587	4
lomustine	0.786	0.00408	0.0882	100	0.472	4
clomifene	0.773	0.00507	0.0402	100	0.509	4
cloperastine	0.743	0.00077	0.0052	100	0.499	6
benzamil	0.731	0.00101	0.0191	83	0.57	6
bromocriptine	0.73	0.00336	0	100	0.494	5
resveratrol	0.696	0.00006	0.0931	77	0.494	9
chlorcyclizine	0.677	0.00312	0.015	100	0.492	6
thioridazine	0.548	0	0.1918	65	0.386	20
prochlorperazine	0.49	0.00038	0.1553	56	0.275	16
monorden	0.467	0.00008	0.0642	72	0.307	22
trifluoperazine	0.46	0.0011	0.2885	62	0.367	16
fluphenazine	0.391	0.00565	0.2798	55	0.285	18
sirolimus	0.352	0.00002	0.2048	63	0.267	44
LY-294002	0.337	0	0.2617	55	0.299	61
trichostatin A	0.319	0	0.6825	63	0.217	182
rosiglitazone	-0.467	0.00232	0.0124	57	-0.249	14
hydroquinine	-0.774	0.00537	0.0142	75	-0.291	4
cinchonine	-0.793	0.00368	0.0556	75	-0.343	4
fursultiamine	-0.801	0.00308	0.0051	50	-0.311	4
quinpirole	-0.931	0.00002	0	100	-0.592	4

Supplementary Methods

Connectivity map (build02)

Briefly, the DEGs identified in our transcriptome were converted in microarray probe ID in Affymetrix HG U133A by ensembl Biomart conversion tools [2] (http://uswest.ensembl.org/biomart/martview). The converted pro ID were reversely queried to the connectivity map [3, 4] (version build 02, https://portals.broadinstitute.org/cmap/), and the results were output by form of permuted results. For selection, criteria of 1) the number of repeat experiments to more than 4 times (n ≥ 4), 2) the proportion of effective rate to ≥ 50% (percent ≥ 50), and 3) P-value ≤ 0.05 were applied. Compound were then arranged by their enrichment score in descending order (Supplementary Table S4).

Supplementary Reference

1. JC O. Venny. An interactive tool for comparing lists with Venn's diagrams. 2007-2015.
2. Howe KL, Achuthan P, Allen J, Allen J, Alvarez-Jarreta J, Amode MR, et al. Ensembl 2021. Nucleic acids research. 2021;49(D1):D884-d91.
3. Lamb J, Crawford ED, Peck D, Modell JW, Blat IC, Wrobel MJ, et al. The Connectivity Map: using gene-expression signatures to connect small molecules, genes, and disease. Science (New York, NY). 2006;313(5795):1929-35.
4. Lamb J. The Connectivity Map: a new tool for biomedical research. Nature reviews Cancer. 2007;7(1):54-60.
